# The Maternal Transcriptome of the Crustacean *Parhyale hawaiensis* Is Inherited Asymmetrically to Invariant Cell Lineages of the Ectoderm and Mesoderm

**DOI:** 10.1371/journal.pone.0056049

**Published:** 2013-02-13

**Authors:** Peter Nestorov, Florian Battke, Mitchell P. Levesque, Matthias Gerberding

**Affiliations:** 1 Max Planck Institut für Entwicklungsbiologie, Tübingen, Germany; 2 Center for Bioinformatics, University of Tübingen, Tübingen, Germany; University of Otago, New Zealand

## Abstract

**Background:**

The embryo of the crustacean *Parhyale hawaiensis* has a total, unequal and invariant early cleavage pattern. It specifies cell fates earlier than other arthropods, including *Drosophila*, as individual blastomeres of the 8-cell stage are allocated to the germ layers and the germline. Furthermore, the 8-cell stage is amenable to embryological manipulations. These unique features make *Parhyale* a suitable system for elucidating germ layer specification in arthropods. Since asymmetric localization of maternally provided RNA is a widespread mechanism to specify early cell fates, we asked whether this is also true for *Parhyale*. A candidate gene approach did not find RNAs that are asymmetrically distributed at the 8-cell stage. Therefore, we designed a high-density microarray from 9400 recently sequenced ESTs (1) to identify maternally provided RNAs and (2) to find RNAs that are differentially distributed among cells of the 8-cell stage.

**Results:**

Maternal-zygotic transition takes place around the 32-cell stage, i.e. after the specification of germ layers. By comparing a pool of RNAs from early embryos without zygotic transcription to zygotic RNAs of the germband, we found that more than 10% of the targets on the array were enriched in the maternal transcript pool. A screen for asymmetrically distributed RNAs at the 8-cell stage revealed 129 transcripts, from which 50% are predominantly expressed in the early embryonic stages. Finally, we performed knockdown experiments for two of these genes and observed cell-fate-related defects of embryonic development.

**Conclusions:**

In contrast to *Drosophila*, the four primary germ layer cell lineages in *Parhyale* are specified during the maternal control phase of the embryo. A key step in this process is the asymmetric distribution of a large number of maternal RNAs to the germ layer progenitor cells.

## Introduction

The establishment of germ layers is a major decision about future cell fates and organs that all embryos take early in development but reach in different ways. So far, progress has been made in understanding germ layer formation in model organisms that represent different phyla. This progress, however, has been slower in addressing the diversity found among non-model organisms and within phyla. For the phylum of arthropods, studies in the fruit fly *Drosophila* are leading the way. Analysis of early *Drosophila* development has characterized many of the genes and interactions of their products responsible for the specification of the germ layers and the germ line. The first studies used forward genetic screens to identify Dorsal protein as a primary asymmetry factor upstream of the specification of germ layers [1], [2]. Microarrays and large-scale *in situ* screens uncovered a small number of additional zygotic RNAs that are differentially distributed along the dorso-ventral axis [3], [4]. Traits found in the development of *Drosophila* do not apply to arthropods in general, as embryos of arthropods show highly divergent traits, especially during early stages and germ layer formation. The scope of arthropod developmental diversity is becoming increasingly obvious from the growing number of studies of embryonic development across different arthropod taxa [5], [6]. The study of species with divergent modes of embryonic development can reveal divergent molecular programs [5], [7], [8], [9].

The embryo of *Parhyale* shows a germ layer formation by an invariant early cell lineage. In the first three zygotic divisions, eight cells are generated that differ in their morphology. Each of these cells is programmed to contribute exclusively to only one of the germ layers [10],[11]. This fate restriction for germ layers has no correspondence in *Drosophila* but resembles the situation found in *Caenorhabditis elegans* and *Ciona* [12]. In *Paryhale*, however, it takes place earlier than in *C. elegans* and *Ciona* and it is the earliest establishment of germ layer restriction known in animals. Previous studies in *Parhyale* on a few candidate genes showed no early spatial restriction of RNA among the blastomeres of the 8-cell embryo [13],[14],[15]. One of the limitations of emerging model organisms is the lack of genome sequence data and gene annotations. In fact, there are currently only 49 entries in NCBI’s GenBank for *P. hawaiensis* and the only crustacean organism that has been sequenced and the data has been made publicly available is the water flea *Daphnia pulex* [16]. A genomic BAC library for Parhyale has been generated and partially sequenced in attempt to uncover *Parhyale*’s 3 Gb genome [17]. Recently, two independent laboratories applied next generation sequencing to analyse the developmental transcriptome of *Parhyale* through *de novo* assembly and homology searches [18],[19]. Both studies point to a highly complex transcriptome, which raises the need of a transcriptomics approach to identify the major genes controlling developmental processes such as lineage specification in the embryo. Therefore, the current study set out to address a potential temporal and spatial restriction of RNAs using a high-density oligonucleotide microarray. Specifically, we asked what portion of the known ESTs (Expressed sequence tags) correspond to maternal transcripts; what portion of these maternal transcripts is distributed differentially among mesoderm and ectoderm progenitors; and whether a function for maternal RNAs in mesoderm and ectoderm formation can be shown. By combining the microarray with immunocytochemistry, *in situ* hybridization, and gene knockdown experiments, we demonstrate that 10% of the 6386 targets are maternal transcripts and that more than 10% of these maternal transcripts are inherited differentially to the progenitors of the four germ layers. In addition, we show for two asymmetrically distributed maternal RNAs that their knockdown has an effect on embryonic development.

## Results

### Custom Gene Expression Microarray for *Parhyale hawaiensis*

The current study aimed at a large-scale identification of transcripts involved in germ layer specification during early development of *Parhyale hawaiensis*. For this purpose we used the existing *Parhyale* EST libraries and the Agilent eArray platform to design a high-density microarray in the 8×15 k format, i.e. eight arrays on a single slide and each array consisting of 15744 probe 60-mer DNA oligonucleotides. The GEO accession number for the microarray platform is GPL16208. The microarray was based on 9042 EST sequences, which were assembled in 6386 contigs (including singletons). We employed a bioinformatics approach to map all probes to their corresponding target (excluding cross-hybridizing probes) and then calculated the average expression values, fold-change values and statistical analysis taking into account the number of probes per target. Detailed explanation of EST contig assembly, probe mapping and cross-hybridization analysis is provided in the Material and Methods section. Furthermore, for 16 genes of interest we designed multiple probes, which were spotted a total of 742 times on the chip (*Ph-vasa*, *Ph-armadillo*, *Ph-brachyury, Ph-gata, Ph-midline, Ph-nanos, Ph-PL10, Ph-tinman* and several *Wnt* homologs). We also designed both sense and antisense probes for a set of 181 ESTs, which allowed us to determine the background expression signal. Finally, the array includes 536 manufacturer control probes that were used for quality control (integrated in Agilent’s Feature Extraction software).

### Immunohistochemical Assay Indicates an Onset of Zygotic Transcription After Germ Layer Specification 690 of the 1494 Expressed Genes are Maternally Enriched

**Figure 1:**
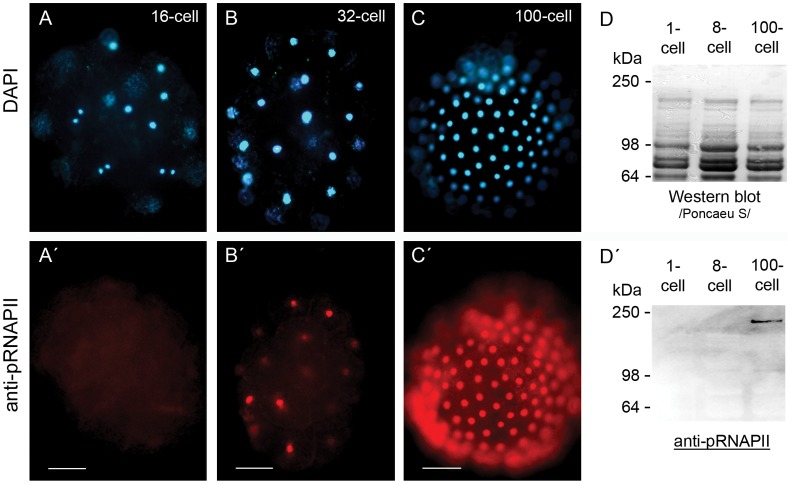
Immunodetection of early embryonic transcription. Immunohistochemical detection of active RNAPII was performed with a commercially available monoclonal antibody against the Ser2 phosphorylated CTD of RNAPII (H5) and an Alexa 446 conjugated secondary antibody. (A-C) DAPI stained nuclei of a 16-cell stage embryo (A), 32-cell stage embryo (B) and a 100-cell stage embryo (C). An antibody signal was not detected in 16-cell stage embryos (A′). First signs of active transcription were visible in 32-cell stage embryos (B′) and fully active transcription was detected in 100-cell stage embryos (C′). Scale bar corresponds to 100 µm. In order to confirm the whole mount immunostainings, we performed a Western blot with protein extracts from 1-cell, 8-cell and 100-cell embryos (**D**). The 220 kDa phosphorylated RNAPII was detected only in the 100-cell stage protein extract (**D**′).

In a very wide range of eukaryotic species, the initial steps of embryonic development occur under maternal control, while the zygotic genome remains transcriptionally silent [20]. The embryo takes over the control of development in the process of maternal to zygotic transition (MZT), when the zygotic genome is activated and maternally provided determinants are gradually degraded. We used an antibody against the Ser2-phosphorylated RNAPII to detect transcription in early *Parhyale* embryos and determined the time point of maternal-zygotic transition (Figure 1). A signal was first detected in some cells of 32-cell-stage embryos, indicating the start of MZT, possibly with a minor wave of genome activation, preceding the major genome activation (Figure 1B, B′). A uniform signal in all cells was detected in 100-cell-stage embryos, suggesting the start of the zygotic control of embryonic development (Figure 1C, C′). Furthermore, we performed an immunoblot and detected activated RNAPII in protein extracts from 100-cell stage embryos but not in 1- or 8-cell embryos, which confirmed the observed immunohistochemical (IHC) staining (Figure 1D, D′). These results suggest that germ layer progenitors at the 8-cell stage of *Parhyale* are specified under maternal control, while the zygotic genome is still transcriptionally silent.

**Figure 2:**
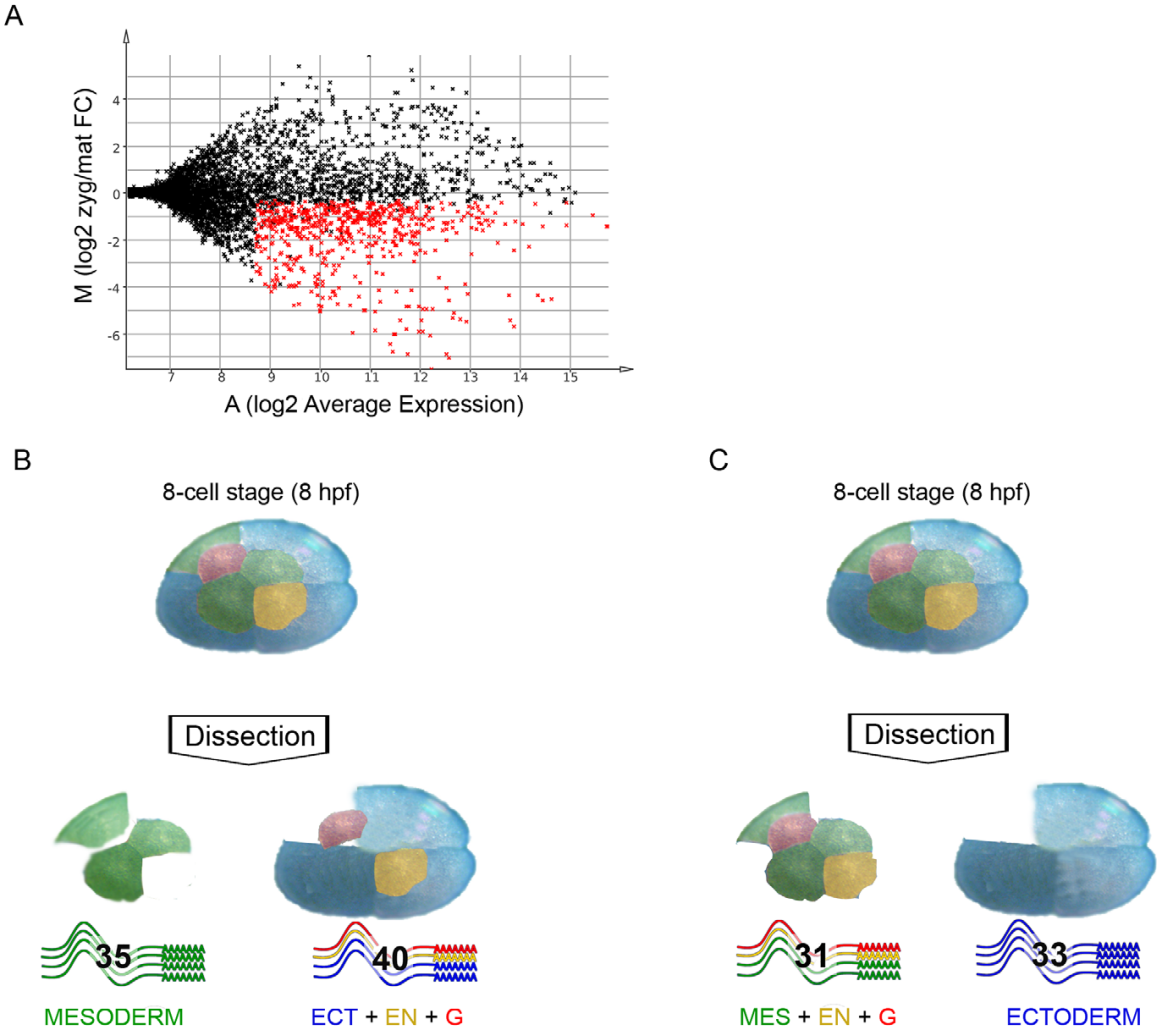
Microarray analysis of *Parhyale* embryos. Labelled RNA pools from transcriptionally inactive embryos (1- to 4-cell stage) and RNA pools from transcriptionally active embryos (germ band and limb bud stages) were hybridized together in a two-colour microarray experiment. (A) The MA plot uses the logarithmic fold-change value “M” as the y-axis and the logarithmic average expression value “A” as the x-axis, where: M = log2(zyg/mat), A = 1/2(log2(mat)+log2(zyg)), mat = signal intensity for transcriptionally inactive embryos, zyg = signal intensity for transcriptionally active embryos. The red data points represent the 690 unique maternally enriched RNAs, which constitute more than 10% of all unique gene sequences on the microarray. (B, C) The 8-cell embryo consists of four macromeres and four micromeres, each of them invariantly contributing to only one of the germ layers or the germ line. The three blue-coloured blastomeres give rise to ectoderm, the green-coloured blastomeres establish mesoderm, the yellow-coloured micromere is the progenitor of endoderm and the red-coloured cell contributes to the germ line. Blastomeres of the 8-cell stage were separated from each other by dissection and used for preparation of labelled cRNA for a two-coloured microarray experiment. The comparison between RNA from mesoderm progenitors and the rest of the 8-cell stage embryo (progenitors for ectoderm, endoderm and germline) resulted in the identification of a total of 129 asymmetrically localized RNAs. In the first experiment we found 35 RNAs overrepresented in the mesoderm progenitors transcript pool and 40 RNAs that were underrepresented. The second experiment approach revealed 33 transcripts enriched in ectoderm progenitors and 31 others that were predominantly found in the RNA pool from mesoderm, endoderm and germline progenitors. The four groups of asymmetrically localized RNAs from the two experiments add up to 139 because 10 of the RNAs were found in both experiments (see Table S4).

As evident from the IHC assay and the immunoblot (Figure 1), there is no zygotic transcription up to the 8-cell stage. Therefore, RNA pools isolated from these stages were considered as maternally provided transcripts only. Total RNA was isolated from the first three stages of embryonic development (1- to 4-cell stage respectively) and from transcriptionally active embryos (100-cell germ disc and germ band stage embryos). The labelled cRNA was hybridized to the microarray as described in Figure S1. Data analysis of the microarray hybridization revealed the maternally enriched transcripts in respect to the zygotic transcriptome (Table S1, GEO accession number GSE41885). By setting the cut-off for the adjusted p-value at 0.05, we identified 1523 targets enriched in the early stages of development and 1278 differentially expressed in transcriptionally active embryos. We made the analysis more stringent by introducing an additional cut-off for the average expression value (A-value). For this purpose we determined the mean A-value of the internal negative controls (antisense targets), and set the cut-off three standard deviations away from the mean (at an A-value of 8.8, corresponding to about 450 relative fluorescent units in a range from 0 to 65000). This led us to a group of 1494 genes, which we defined as being expressed in early and/or late embryos. A striking difference between the maternal and zygotic transcriptomes was observed, since 1258 of the 1494 expressed genes were enriched in one of the RNA pools (Figure 2A). 690 of the target genes were maternally enriched and 568 were enriched in the zygotic pool.

**Figure 3:**
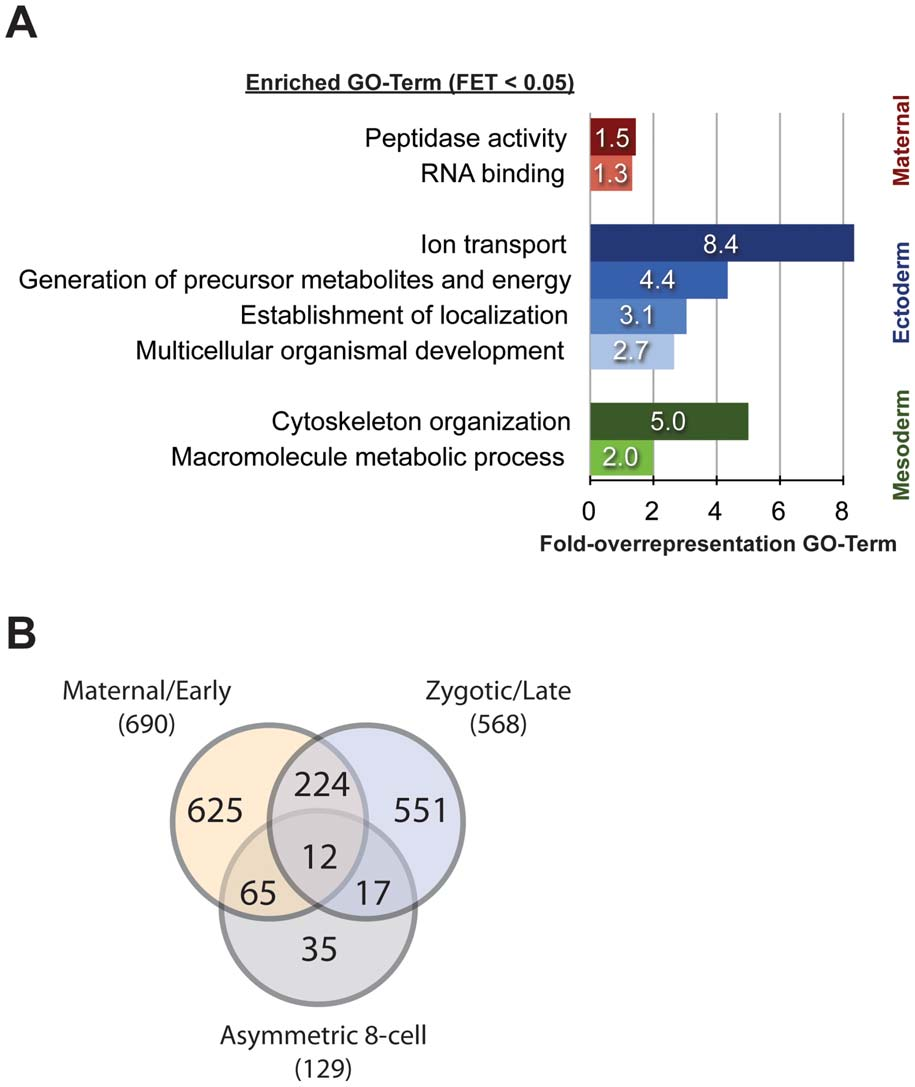
Asymmetrically distributed RNAs at the 8-cell stage of *Parhyale*. (A) GO enrichment analysis for the asymmetrically distributed RNAs at the 8-cell stage of *Parhyale*, as well as for the maternally provided transcripts. The bars represent the GO-term fold-overrepresentation (percent of sequences in the analysed group associated to a certain GO-term over the percentage for this GO-term in the reference group) for significantly overrepresented GO-terms (Fisher’s Exact Test P-value less than 0.05) within the 690 maternally enriched targets (red bars), the 35 RNAs enriched in mesoderm progenitors (green bars) and the 33 ectoderm progenitor transcripts (blue bars). (B) A cross-correlation was performed between the data sets for (1) asymmetrically distributed RNAs at the 8-cell stage and (2) differentially expressed genes in transcriptionally inactive (pre-MZT) and transcriptionally active embryos. The left circle of the Venn diagram contains the 690 maternally enriched RNAs, 65 of which are asymmetrically distributed at the 8-cell stage (intersection with lower circle). The intersection of the left and right circles includes 236 targets expressed at equal levels during early and late embryonic development, with 12 being asymmetrically localized at the 8-cell stage. The right circle consists of the 568 genes predominantly expressed from the zygotic genome at later embryonic stages, including the 29 asymmetric at the 8-cell stage. There are 35 low-expressed RNAs, which are still showing statistically significant changes between the blastomeres of the 8-cell.

We used sequence homology analysis to elucidate the possible function of the maternally provided gene products. By using the blast2go platform for annotation and gene ontology (GO) analysis of microarray data [21], we annotated and assigned homology-based GO-terms for the maternally provided RNAs. GO-enrichment analysis did not show any overrepresented GO-terms within the 690 maternally provided genes compared to the 1494 expressed ones. However, we found peptidase activity (GO:0008233) and RNA binding (GO:0003723) to be enriched when we compared the maternal transcriptome to the whole set of 6386 targets (Figure 3A and Table S5).

### 129 RNAs are Distributed Asymmetrically among the Progenitors for the Germ Layers of *Parhyale*

We performed two separate experiments, where 8-cell stage embryos were dissected and subsets of blastomeres were used for the generation of labelled complementary RNA for microarray hybridization (see Materials and Methods and Figure S1). Our experimental design was based on the described invariant cell lineage of *Parhyale* [10] and aimed at the differential transcriptome comparison between (1) the ectoderm progenitors and the rest of the 8-cell embryo, and (2) the mesoderm progenitors and the remaining 5 blastomeres (see colour-coded representation of the experimental design in Figures 2B and 2C). We observed good correlation across replicates in both experiments (Figure S2). Differential gene expression analysis with an adjusted p-value cut-off of 0.05, led to the identification of 35 RNAs enriched in the mesoderm progenitors transcript pool and another 33 RNAs that were predominantly found in the ectoderm progenitors transcriptome (Tables S2 and S3; summarized results in Table S4, GEO accession numbers GSE41886 and GSE41887 for Ect_vs_rest and Mes_vs_rest respectively). A further 61 unique transcripts were identified as enriched in either one of two larger subsets of blastomeres that were used in the two arrays. One pool comprised the non-ectodermal cells, i.e. the three mesoderm progenitors plus endoderm plus germline progenitor cells on the one hand; the second pool comprised the non-mesodermal cells, i.e. the three ectoderm progenitors plus endoderm plus germline progenitor cells on the other hand Next, we annotated the 129 genes and performed GO-term enrichment analysis in respect to the GO-term distribution of the 690 maternally provided genes (Figure 3A and Table S5). Interestingly, we found that the mesoderm- and ectoderm-enriched transcripts are associated with different GO-terms. In the mesoderm progenitors, we found an enrichment of genes coding for cytoskeleton organization (GO:0007010) and macromolecule metabolic processes (GO:0043170). The ectoderm progenitor genes are involved in the establishment of localization (GO:0051234), ion transport (GO:0006811), generation of precursor metabolites and energy (GO:0006091) and multicellular organismal development (GO:0007275). Our experimental design suggests redundant findings in the two experiments, i.e. if a gene is highly enriched in the ectoderm progenitor pool, ideally it would come up in the corresponding RNA pool in both experiments. Indeed, we found 9 of the 33 ectoderm-enriched genes in the corresponding blastomere pool from the other experiment, i.e. the ectoderm, endoderm and germline blastomeres. Furthermore, we found *Ph-vasa*, a helicase-coding RNA associated with the germline, to be enriched in the pool of mesoderm, endoderm and germline blastomeres. This enrichment is in agreement with the previously described expression of the gene [15].

### Cross-correlation Analysis Indicates that Half of the Asymmetrically Localized RNAs get Dramatically Reduced at the Maternal-zygotic Transition

With respect to the levels of zygotic gene expression, the 129 RNAs that were identified in the screen for differentially distributed transcripts belong to one of three possible groups of maternally provided RNAs: 1) maternally provided RNAs that are not transcribed zygotically (or transcribed at very low levels); 2) maternally provided, zygotically transcribed RNAs with constant levels of transcription throughout development; 3) maternally provided, zygotically transcribed RNAs with higher levels during the zygotic control of embryogenesis. We performed cross-correlation analysis with the generated microarray data sets and allocated the 129 asymmetrically distributed RNAs in one of these three groups (Figure 3B, Table S4). Half of the 129 RNAs belong to the set of transcripts enriched in early embryos and therefore fall into the first group of maternally provided transcripts. Furthermore, most of these 65 genes display a very high fold-change-value (early vs. late expression), reaching 180-fold for *zaFBplate1_H17_073.ab1*, which is an indication for high levels in early stages and almost full depletion of the RNA in later stages. Gene annotation revealed genes coding for zinc-finger proteins, nucleic acid binding proteins and serine proteases. *Ph-vasa* and a novel small glycine rich protein gene *Ph-cg0548* (microarray target: zamn02_K22_085.ab1) are also highly upregulated in the maternal transcriptome, with *Ph-vasa* showing a 10-fold enrichment and *Ph-cg0548* 50-fold. The second group of maternally provided transcripts, expressed at equal levels during later stages, is represented by 12 of the 129 asymmetrically distributed RNAs. The 17 transcripts that were predominantly found in the zygotic transcript pool (i.e. the third group of maternally provided RNAs) are predominantly coding for proteins involved in metabolism. Finally, 35 RNAs have not been assigned to one of the three groups since they have low average expression values in the Early_vs_late experiment. Nevertheless, 14 of these 35 RNAs are more than 1.5-fold upregulated in the maternal transcript pool and show statistically significant changes for their expression in the blastomeres of the 8-cell stage.

### Asymmetrically Distributed RNAs Identified by the Microarray are Validated by in situ Hybridization

**Figure 4:**
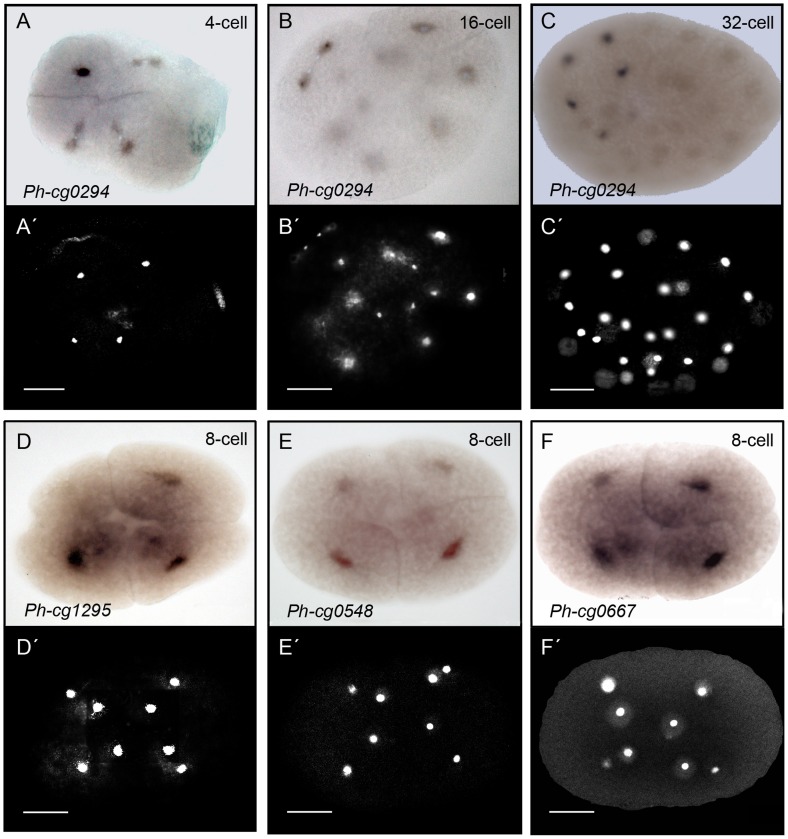
Whole mount *in situ* hybridization analysis of asymmetrically distributed RNAs at the 8-cell stage of *Parhyale*. Shown here four out of the six RNAs that generated signal. The RNA is detected in the small perinuclear cytoplasm at different signal intensities. (A-C) Bright field views and (A′–C′) corresponding DAPI stained images of three different embryos stained for *Ph-cg0294* (microarray target: Contig0106) and DNA. The cytoplasmic *in situ* signal (dark) is concentrated in the precursor of the *Mav* cell at the 4-cells stage shown in (A) (*Mav* is the progenitor of anterior visceral mesoderm, 2^nd^ quadrant of image, see Figure 2B). The nucleus of the cells is in the centre (A′), surrounded by a small region of cytoplasm (*in situ* staining only in cytoplasm). The remaining volume of the blastomeres is yolk-rich. (B, B′) An embryo entering the 16-cell stage and the *Mav* cell finishing the process of division. The *in situ* signal does not colocalize with the nuclei of the daughter cells of *Mav*, but is rather cytoplasmic. Weak signal is also visible in the daughter cells of *El* (progenitor of left ectoderm, 1^st^ quadrant). (C, C′) A 32-cell stage embryo with strong cytoplasmic staining in four cells (the lineage of *Mav*) and weaker signal in a fifth cell. (D, D′) An 8-cell stage embryo showing the expression pattern of *Ph-cg1295* (microarray target: zaFBplate1_E16_060.ab1). A strong signal was detected in the progenitors of ectoderm and weaker signal was visible in two of the micromeres. (E, E′) The WISH analysis for *cg0548* (microarray target: zamn02_K22_085.ab1) shows weak staining in the macromeres but not the micromeres of the 8-cell stage. (F, F′) WISH analysis on 8-cell stage embryos for cg0667 (microarray target: zamn01_E08_028.ab1) showed stronger signal in the ectoderm macromeres, weaker signal in the mesoderm macromere and no signal in the micromere progenitors. Scale bar corresponds to 100 µm.

In order to validate our results, we performed whole-mount *in situ* hybridization (WISH) for ten genes that were identified as asymmetrically distributed either in the ectoderm progenitors transcriptome or in the RNA pool of mesoderm, endoderm and germline progenitors. These targets were among the top 20 showing the highest fold-change values in our preliminary microarray analysis that was based solely on the EST sequences and not accounting for multi-spotted and multi-probe targets. The cells at the 8-cell stage of *Parhyale* are different from blastomeres in *Xenopus,* or *C. elegans* as the area and volume of the nuclei and cytoplasm are small in terms of total volume of the large cells. The cells are packed with yolk and the nuclei are located at the centre and surrounded by only a small perinuclear cytoplasm. Six of the ten genes we investigated showed an *in situ* hybridization (ISH) signal in the perinuclear cytoplasm. These RNAs showed an elevated signal in the expected subset of blastomeres (Figure 4 and Table S6). An expression of the other four analysed genes was not detected in the stained embryos. The strongest ISH signal was observed for *Ph-cg0294* (microarray target: Contig0106) in the lineage of the visceral mesoderm progenitor cell (*Mav*-cell, Figure 4A–C). *Ph-cg0294* was asymmetrically distributed already at the 4-cell stage (Figure 4A, A′). In the 8-cell stage embryo, weak cytoplasmic signal was observed in most of the cells but *Mav* showed the strongest staining (data not shown). In the 16- and 32-cell stage embryos the signal was detected in the cytoplasm of the *Mav*-lineage (i.e. two cells at 16-cell stage and four cells at 32-cell stage, Figure 4B–C). The genes, *Ph-cg1295, Ph-cg0548* and *Ph-cg0667* showed a signal in the macromeres. Either the signal was strongest for the ectoderm-macromeres, as in the case of, *Ph-cg1295* and *Ph-cg0667* (Fig. 4D and 4F) or it showed up for all macromeres, as in the case of *Ph-cg0548* (Figure 4E). Next, we performed knockdown experiments and found phenotypes for *Ph-cg1295* and *Ph-0548* described below.

### Cell-cycle Defect in 4-cell Embryos after Knockdown of the Localized RNA *cg1295*

**Figure 5:**
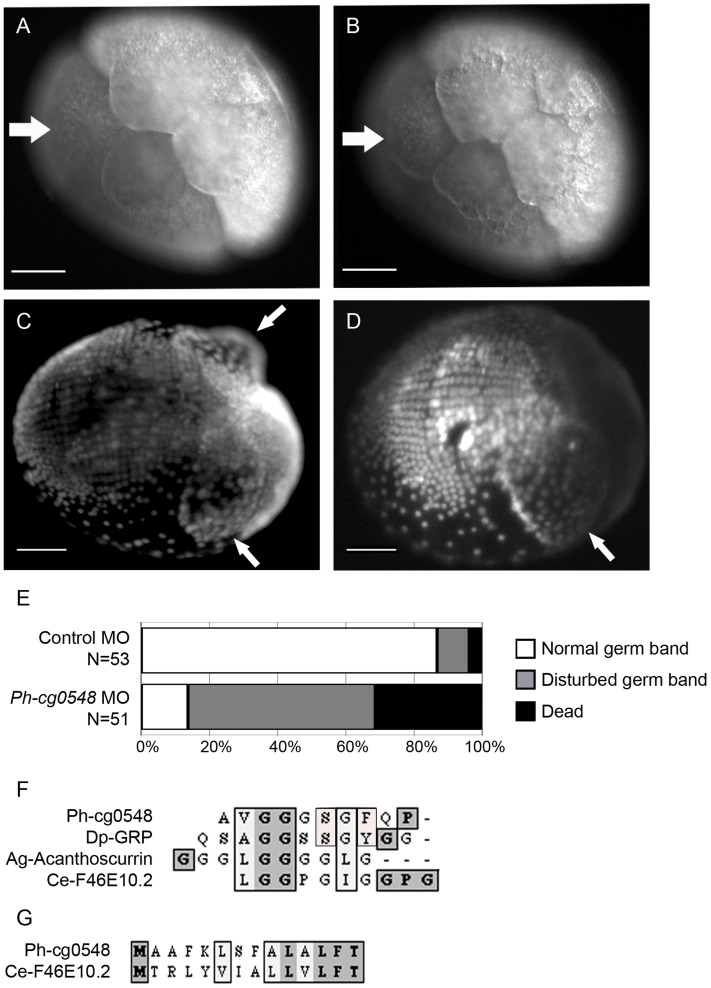
Functional analysis of *cg129*5 and *cg0548* by knockdown experiments. (A, B) *In vi*vo imaging of an siRNA-injected embryo (200 µM Stealth-RNA, 1-cell stage microinjection) imaged at 6 hpi (A) and 6,5 hpi (B). The white arrow in (A) indicates the site of the missing micromere cell after incomplete third cleavage. The same embryo was imaged 30 min later (6,5 hpi, B). The white arrow indicates the micromere cell that has just formed after a delayed division of one blastomere cell from the 4-cell stage. The changing shape of the other blastomeres at 6,5 hpi is a sign of initiated fourth cleavage (from 8- to 16-cell stage). Scale bar corresponds to 100 µm. (**C**) A ventro-lateral view of a DAPI-stained, control morpholino injected embryo fixed at 5 dpi. The white arrows indicate the position of the midgut anlagen (disc-shape structures). The ectodermal cell rows are clearly visible on the posterior ventral side of the embryo. (**D**) A ventro-lateral DAPI view of a anti-*cg0548* morpholino-injected embryo fixed at 5 dpi. The white arrow indicates the single disc-shape structure in the anterior part, whereas the second one is missing. (**E**) Phenotype scoring and survival of control and anti-*cg0548* morpholino-injected embryos at 5 dpi. More than 80% of the control embryos show normal germ band formation. In contrast, only 15% of the anti-*cg0548* morpholino-injected embryos were developing normal and 30% were already dead at the observed time point. (**F**) The *cg0548* RNA is coding for a small glycine-rich protein. The protein sequence includes 11 tandem repeats of the sequence Ala-Val-Gly-Gly-Gly-Ser-Gly-Phe-Gln-Pro (AVGGGSGFQP). Possible homologs of Ph-cg0548 were found in a wide range of organisms (from the closely related *Daphnia pulex* to the worm *Caenorhabditis elegans*). These proteins have short amino acid sequence (several hundred residues) and glycine-rich tandem repeats. Alignment of the repetitive element shows that *Parhyale* and *Daphnia* share the highest similarity, including a serine and an aromatic residue (F/Y). The position of the proline residue is shared between *Parhyale* and *C. elegans*. (**G**) Sequence alignment of the non-repetitive N-terminal part of the protein shows that *C. elegans* and *Parhyale* both have a stretch of aliphatic residues and a conserved phenylalanine, followed by a threonine.

For the knockdown screen, we picked the six genes that had been validated by WISH. We were able to observe a phenotype for two of these genes – *Ph-cg1295* and *Ph-cg0548*. *Ph-cg1295* RNA (microarray target: zaFBplate1_B21_095_ab1) is enriched in the three ectoderm progenitors at the 8-cell stage, as shown in two independent microarray experiments and by WISH. The available partial cDNA sequence for *Ph-cg1295* does not share homology with annotated genes from the NCBI database. RACE-PCR (Rapid-amplification of cDNA ends by PCR) did not give any results with several different sets of primers and templates. Since the position and the surrounding sequence of the start codon are unknown, we used siRNA-mediated knockdown to study the function of *Ph-cg1295*. 1-cell embryos were injected with Stealth-RNA (custom siRNA from Invitrogen) and their development was recorded over the next 14 days until hatching. In healthy embryos, the transition from 4- to 8-cell stage occurs by simultaneous asymmetric division of all four cells. In the knockdown experiment one of the cells at the 4-cell stage divided later than the other three, which was observed for 30% of the siRNA-injected embryos (N = 283). Due to its size and position, the affected cell was identified as either the precursor of visceral mesoderm and germline or the precursor of posterior ectoderm and endoderm (Figure 5A, B). The recorded delay was around 30 minutes, after which the cell divided asymmetrically. At this time point the other (unaffected) blastomeres had already entered the fourth cleavage and divided within 10–15 minutes after the missing cell had appeared. In summary, the phenotype could be described as the existence of a 7-cell stage and an abnormally short 8-cell stage. No defects were observed after the fourth cleavage and the embryos hatched normally. The efficiency of the knockdown following the Stealth-RNA injection was tested by semi-quantitative PCR on cDNA from treated and control embryos, which confirmed the absence of the target RNA (Figure S3).

### Disrupted Mesoderm Formation after Knockdown of the Localized RNA for a Small Glycine-rich Protein *Ph-cg0548*

The *Ph-cg0548* RNA (microarray target: zamn02_K22_095) was predominantly found in the ectoderm progenitor cells at the 8-cell stage both in the WISH and microarray experiments. The 450 bp partial cDNA sequence of the corresponding EST contains the 3′-end of the transcript. 5′-RACE was performed in order to obtain the full length open reading frame of the mRNA (ORF). This PCR-based experiment resulted in the cloning of a second fragment corresponding to *Ph-cg0548*, which contained a potential start codon. By analysing the partial 954 bp cDNA, an ORF was identified that codes for a 196 amino acid protein. The protein contains 11 tandem repeats of the following sequence: Ala-Val-Gly-Gly-Gly-Ser-Gly-Phe-Glu-Pro. BLASTx and BLASTp searches were performed in an attempt to identify homologs of the Ph-cg0548 protein. Because of the high glycine-content and the repeats, the BLAST search returned a list with members of the bacterial PE-PGRS family (Pro/Glu domain containing polymorphic GC-rich repetitive sequences). These proteins contain multiple tandem repeats of Gly-Gly-Ala or Gly-Gly-Asn and consist of around 2000 amino acids [22]. Ph-cg0548 does not have a PE-domain and is much shorter than the PE-PGRS proteins. A literature search led to the finding of Acanthoscurrin. This small antimicrobial protein was found in tarantula spiders (*Acanthoscurria gomesiana*) and consists predominantly of glycine residues [23]. The amino acid sequence alignment of *Ph-cg0548* shows similarity not only to the glycine-rich part but also to the N-terminal part of Acanthoscurrin, where the signalling sequence of the spider peptide is found (Figure 5F). The search for other potential homologs revealed a short protein in *C. elegans* (consisting of 139 amino acids), which shares homology with the N-terminal part of Ph-cg0548 as well as with the glycine-rich domain (gene F46E10.2 on chromosome V, Figure 5F, G). The *C. elegans* gene coding for the small glycine-rich protein is expressed only in embryos and the transcript is differentially distributed (clone YK572b7, The Nematode Expression Pattern Data Base, v. 4.0, Genome Biology Lab, National Institute of Genetics, Mishima, Japan).

We injected antisense morpholino oligonucleotide into 1-cell embryos and followed embryogenesis until hatching. The first phenotype was observed at the late 100-cell stage (germdisc formation and condensation, 15–20% of embryonic development, 3–4 dpi) in a small number of morpholino-injected embryos. Affected embryos showed abnormal germdisc condensation and formation of multiple clusters of cells along the surface of the embryo. These embryos could not develop further and died within the next 1–2 days. The most abundant phenotype was visible around 4–6 dpi at the germband formation and elongation stage (i.e. 25–35% of embryonic development, 4–6 dpi). The laterally positioned midgut anlagen become visible as disc-shaped structures in healthy germ band embryos. In contrast, the morpholino-injected embryos had only one or did not form any disc-shaped structures visible in living embryos. Furthermore, the ectodermal cell row formation was disarranged and cells were distributed randomly as was observed in fixed, DAPI-stained embryos (Figure 5C, D). Eventually, all of these embryos ceased their embryonic development and died within 2–3 days (Figure 5E). Only 15% of the embryos at 6 dpi could form appendages and develop further. After following the complete embryonic development, only 10% of the embryos injected with a morpholino against *Ph-cg0548* hatched, whereas the control-injected embryos showed almost 80% survival rate.

## Discussion

The current study in *Parhyale* found a differential inheritance of maternal RNAs among the eight progenitors of the germ layers. Our study shows that 690 of the 6386 RNAs included in this analysis are detected in early, transcriptionally inactive embryos and are therefore maternally provided. 129 of these maternal transcripts show up as differentially distributed to either ectoderm or mesoderm progenitors. This represents a molecular strategy for the early embryo that is divergent from the situation in *Drosophila*. Maternal RNAs in *Drosophila* are localized along the anteroposterior axis. Remarkably, there is no maternal RNA in *Drosophila* that is localized dorsally or ventrally [3]. The ectoderm and mesoderm are specified zygotically according to their dorsoventral position [4]. Comparing *Parhyale* to *Drosophila* illustrates how much of an early program could change within a phylum such as the Arthropoda, towards more maternal control and towards a germ layer specification by spatial regulation of RNAs. It also highlights a mode of development that seems to utilize differential RNA distribution in a quantitative manner and demonstrates a new arthropod system for the mechanism of cellular polarity [24].

### Transcriptomics Analysis of Germ Layer Formation

This is the first use of a microarray in *Parhyale* and to our knowledge in a crustacean embryo (for use in adult crustaceans and in model organisms, see [25],[26],[27],[28]). The successful adoption of the technique to *Parhyale* allows coverage of a much larger set of RNAs than candidate gene analysis could. The experiment was limited by the coverage of the available ESTs. Nevertheless, the number of ESTs used here and the corresponding 6386 targets can be considered sufficient to present a wide view of early development. To support this statement and to assess what percentage of the whole transcriptome our targets cover, we compared them to transcript assemblies based on RNA-seq experiments that were recently presented by groups from Harvard and Nottingham [18],[19]. As the sequences of the Nottingham group are currently unpublished, we focus here on those of the Harvard group. Unfortunately, while the authors report BLAST annotations for 58.8% of the contigs presented in their study, at the time of writing, these have not been made available online. Using BLAST to map our sequences to those reconstructed from RNA-seq data by the Harvard group (and vice-versa), we find that our EST target sequences cover 25% of their contig sequences. Vice-versa, the Harvard contigs can be mapped to 27% of our sequences. This comparison poses two important questions. Firstly, what is the reason for the relatively low overlap? We assume that one important reason for this is differential expression between our samples and those of the Harvard study, such that both datasets do not cover the complete transcriptomic potential of the organism.

Secondly, the Harvard dataset contains about four times as many contigs as ours. Does this suggest that our dataset only covers a tiny portion of the whole transcriptome? We find that the intersection of the two datasets contains about 4.5 times as many Harvard contigs in relation to our own contigs. This suggests differences in the assembly process as key reason for the discrepancy in numbers. ESTs may have been merged in our contig assembly step, which should have stayed separate, but it may also be due to contigs not being correctly assembled in the Harvard dataset. Without a well-assembled reference genome, there is no final answer to this question.

From the fact that each dataset contains about 75% of unique targets missing from the respective other study, we conclude that the two sets are presenting two different views on the transcriptome, with our dataset focusing on embryonic genes by design. Interestingly, limiting the comparison to the targets we find differentially expressed/distributed between the early and late stage, we find that 91.6% of those have matching sequences in the Harvard library.

If the unequally distributed RNAs are categorized by their gene ontology, there is a correlation between several classes and certain progenitors (Figure 3A and Table S5). This suggests that the progenitors act in distinct ways by possibly utilizing different molecular pathways to achieve cell-fate specification. The current study was able to use gene knockdown to demonstrate a function for two RNAs enriched in either the ectodermal macromeres or all macromeres. Taken together, the asymmetry of many RNAs and the function of at least two RNAs open a first understanding of germ layer formation in *Parhyale*. At the time when the progenitors for the germ layers are specified, the maternal RNAs are still present and zygotic transcription is off. Furthermore, the RNAs show a graded distribution not only along the dorsoventral embryonic axis defined by the dorsal set of non-ectoderm and the ventral set of ectoderm progenitors but also along the anterior-posterior axis defined by the mesoderm progenitors located anteriorly and the ectoderm progenitors located posteriorly. This fits the distribution of the progenitors in space and their unique specification. In *Drosophila*, there is an asymmetry of maternal RNAs only along one of the three axes of the embryo, i.e. along the antero-posterior axis [3]. The number of anterior localized RNAs, including *bicoid*, in *Drosophila* is five. The number of posterior RNAs including germ plasm RNAs exceeds one hundred, hinting at the special use of RNAs to set up the germ line at the posterior end [3],[28]. Within the arthropods, bees that are holometabolous like *Drosophila* use localized maternal RNAs for TGF-signalling to specify axes and germ layers and, in addition, Dpp/Mad and JNK-MAPK signalling has also been implicated for this process [9]. Beyond the phylum Arthropoda, RNA asymmetry along one axis and at one end of the embryo is found in the sea squirt *Ciona* [27]. There, multiple *in situ* screens and a microarray identified RNAs localizing to the posterior pole but no RNAs were found to localize at the anterior pole or along the animal - vegetal axis [22]. In *Xenopus,* maternal RNAs are distributed along a single gradient in the animal-vegetal axis before fertilization and fertilization leads to cortical rotation and differential localization of RNAs of the Wnt-pathway [29],[30],[31]. As far as we can tell, the simultaneous specification of progenitors for the germ layers and the asymmetry of maternal RNAs in *Parhyale* to date is unique not only among the arthropods but among the metazoans.

### How can a Differential Distribution of Maternal RNAs be Generated?

We identified 129 RNAs to be over- or underrepresented in different subsets of the blastomeres of the 8-cell stage. These array data do not tell us how the differential distribution of the RNAs is achieved. An experiment showing how maternal *vasa* RNA starts as ubiquitous message and gets restricted to the germ line, can serve as an example for how an RNA can be localized to a subset of cells [15]. If an artificial 3′UTR of *vasa* RNA is fluorescently labelled and injected into the 8-cell stage, it is cleared from all cells except for the germ line. The 3′ UTR of *vasa* is sufficient to mimic the distribution of the endogenous RNA and its sequence must contain a signal for its restricted stabilization. This mechanism could also produce some of the restricted patterns demonstrated by the array and the WISH. Alternatively, one could imagine other mechanisms being responsible for the patterns found [32]. Local synthesis can be excluded for the case of maternal RNAs. Possible mechanisms are diffusion combined with anchoring or active transport. Diffusion combined with anchoring could generate a differential distribution if the RNAs are asymmetrically localized within a cell and get asymmetrically distributed during cell division. Currently, we have only a single case of asymmetrically distributed RNA during a cell division (Figure 4B) and cannot prove nor rule out anchoring. For active transport, the RNA distribution of the stages prior to the 8-cell stage need to be examined because we cannot envision a scenario of intercellular mRNA transport after cell membranes have established the 8-cell stage. If active transport was used to generate a differential distribution of RNA at the 8-cell stage, this would require the RNAs to be transported into different regions of the 1-cell stage and remain in their regions during cell divisions. Localized RNAs were not observed at the 1-cell stage, excluding active transport as a means to generate asymmetry.

### What is the Driving Force for the Global Changes of Early Development in *Parhyale* as compared to the Ancestral State?

The phylogeny of crustaceans and arthropods is resolved well enough to conclude that the total cleavage and invariant cell lineage of *Parhyale* are secondary traits that derive from an ancestral superficial cleavage and from an ancestral variable cell lineage found in most other malacostracan crustaceans and most insects [33],[34]. This makes it likely that the germ layer specification in *Parhyale* is derived, while it is ancestral in *Drosophila* [3],[8]. The driving force for the global change in *Parhyale* towards a maternal control of germ layer formation could be that the process is implemented after just three divisions and comprises just eight cells while in *Drosophila* it starts after thirteen divisions that generate 6000 cells. The high number of localized RNAs plus the fact that a function could be shown for two out of six RNAs tested in knockdowns can be considered as a clue that RNA distribution exerts a complex control in *Parhyale* on the simultaneous specification of germ layers as well as germ line at the 8-cell stage. A majority of the RNAs analysed here do not have a match by BLAST in cDNAs of other organisms. This may reflect that the proteins coded by the RNAs are unique for *Parhyale* or that the sequence information comprising the EST collection contains many short sequences for which homology is too difficult to determine.

### Conclusion

The current study used for the first time a microarray on a crustacean embryo and determined that more than 1000 of the about 6500 studied RNAs for *Parhyale hawaiensis* are maternally provided, whereas 690 of these are enriched during the early stages of embryonic development. We also found that 129 RNAs are unequally distributed among the progenitors of the germ layers and determined a knockdown phenotype for two of the genes. The results present the first transcriptome analysis of germ layer formation in an arthropod beyond *Drosophila* and reveal remarkable differences between germ layer formation in the fly and *Parhyale*.

## Materials and Methods

### Animals Husbandry, Embryo Collection and Staging

*P. hawaiensis* is a direct-developing marine amphipod with an adult size less than 2 cm. *Parhyale* embryos can be easily injected, cultured and recorded in a Petri dish [7]. Animals were cultured in plastic tubes at 25°C and fed with fish food (Tetra Rubin, Tetra GmbH) or yeast every other day and artificial sea water (ASW) was changed weekly. Embryos were collected from the ventral brood pouch of a mother and classified according to the developmental stages described previously [7]. After collection, embryos were cultured in ASW containing 0,1 µM Geldanamycin (fungicide).

### Embryo Fixation and in situ Hybridization

Embryos from different developmental stages were fixed in 3.7% formaldehyde in filtered artificial seawater for 3–4 hours at room temperature and dechorionated by hand dissection with tungsten wire needles. Fixed embryos were washed with PBS and stored in 100% Methanol at −20°C. These embryos were used after rehydration into PT. Digoxigenin-labelled antisense RNA probes were generated for the genes of interest and used at 1 ng/µL final concentration (see in situ RNA probe synthesis). Whole-mount in situ hybridization was performed as described by Price *et al.* [35]. Embryos were mounted on slides in 70% glycerol and imaged. Stained embryos were stored in 70% glycerol at 4°C.

### Microinjection into 1-cell Stage Embryos

Chemically modified siRNA (custom Stealth-RNA, 25 bp, double-stranded, Invitrogen) and antisense morpholino oligonucleotide (Morpholino-modified DNA oligonucleotide, 25 nt, single-stranded, Gene Tools) were injected into 1-cell embryos as described by Gerberding *et al.* [10]. The concentration of the siRNAs was 200 µM (lyophilized buffered Stealth-RNA was resuspended in DEPC-water to a 1 mM stock solution). The sequence of the two siRNAs against Ph-cd1295 is: 5′-ACGCCUAGAGGAGACAACAGUUGAU-3′ and 5′-CAAAUUCCCUUUACCAUCGUGUUUA-3′. The injected morpholino oligonucleotide was 1 mM (from 5 mM stock solution of lyophilized oligonucleotide) and the sequence is 5′-AGCTTAGTTTGAAGGCAGCCATGTT-3′. Injected embryos were checked regularly (hourly during the first 10 hours after injection, then twice a day until hatching). For detailed observation, embryos were fixed as described above and stained with DAPI. DAPI-images were captured on a Zeiss Axiophot Microscope with an appropriate filter and objective and a Colour-View camera (Olympus). Fixed embryos were stored in PBS at 4°C.

### Immunohistochemistry

Embryos were fixed as described above and used for antibody staining after rehydration into PT (optional: the dehydration step in methanol can be skipped and embryos can be used directly after dechorionation). The staining was performed as described by Patel *et al*. [36]. Embryos were incubated with mouse monoclonal antibody [H5] to RNA polymerase II CTD repeat YSPTSPS (phospho S2) (Abcam, ab24758) overnight at 4°C at a 1∶100 dilution. Secondary antibody was AlexaFluor 546 goat anti-mouse IgG antibody (Invitrogen) at 1∶1000 dilution. Embryos were counterstained with DAPI to visualize nuclei. Fluorescent images were captured on an Olympus IX81 Microscope with an appropriate filter and objective and a Colour-View camera (Olympus).

### *In Situ* RNA Probe Synthesis

DNA plasmids containing the fragment of interest were linearized with an appropriate enzyme to generate templates for antisense RNA synthesis with an appropriate RNA Polymerase (T7, SP6 or T3). Linearization of 5 µg of plasmid was performed for two hours at the optimum temperature for the enzyme used and was subsequently purified using a DNA Clean & Concentrator Kit (Zymo) as described in the kit’s manual. Approximately 1 µg linearized DNA was transcribed *in vitro* and labelled with Digoxigenin (2 µl 5× transcription buffer, 1 µl DIG RNA labelling mix (Roche), 0,5 µl RNA Polymerase, 0,5 µl RNase inhibitor, RNase-free water up to 10 µl). Transcription was performed at 37°C for two hours, followed by analysis of 1 µl on a 1,5% agarose gel. RNA was purified using RNeasy Micro Kit as described in the manufacturer’s manual.

### Reverse Transcription

Reverse transcription was performed with the M-MulV-Reverse Transcriptase and total RNA was isolated as described below. Reaction components and conditions were as described in the RT protocol from Fermentas. cDNA for RACE was synthesized according to the manufacturer’s manuals (GeneRacer Kit, Invitrogen or SMART cDNA Library Construction Kit, Clontech). cDNA was purified with the DNA Clean & Concentrator Kit (Zymo) as described in the manufacturer’s manual. Concentration was measured on a spectrophotometer. The cDNA was used as a template in PCR-based experiments.

### PCR, RACE and Cloning

5′-RACE-PCR for *Ph-cg0548* was performed with 5′-RACE templates synthesized as described above. RACE-specific primers were used for the primary and secondary PCR runs as described in the corresponding manual (GeneRacer, Invitrogen; SMART, Clontech). The gene specific primers used for *Ph-cg0548* were derived from a 420 bp sequence from the EST library. A 650 bp 5′-RACE product was amplified, cloned and sequenced using standard cloning techniques [37]. The resulting 955 bp sequence is shown in.

### Western Blotting

Protein extracts for immunoblot analysis were isolated from whole embryos in different stages of development. Embryos were homogenized in 200 µl deyolking buffer using a pestle and by vortexing. Samples were centrifuged at 300 rcf (2200 rpm) for 5 min. at 4°C. Supernatant was discarded and pellets were washed with 200 µl WB wash buffer. After another 5 min. centrifugation at 300 rcf, pellet was washed with 300 µl WB wash buffer, After centrifugation (300 rcf, 5 min), supernatant was removed and pellet was resuspended in 10 µl distilled water. Protein concentration was measured at this point on a spectrophotometer (280 nm absorbance) and probes were diluted appropriately, so that protein amount is in the same range for all samples. 8 µl of aqueous protein solution were added to 4× SDS loading buffer (Fermentas) and incubated on ice for 15 min. Probes were boiled at 95°C for 5 min and directly run on 8%-Tris-Gly SDS-PAGE at 200V until the 200 kDa band of the pre-stained protein ladder (SeeBlue Plus2, Invitrogen) reached the middle of the resolving gel. Electroblotting of the gel to a nitrocellulose membrane was performed at 4°C for 70 min at 200 mA. The blotted membrane was stained with 0,2% Ponceau S in 5% acetic acid for 5 min and subsequently imaged with a camera. Membrane was blocked with 5% BSA in TBST for 60 min. Primary mouse anti-pRNAPII (ab24758) antibody was used in a 1∶250 dilution and was incubated with the membrane overnight at 4°C. Secondary peroxidase conjugated anti-mouse IgG antibody was used in a 1∶2000 dilution and incubated with the membrane for 60 min. at room temperature. Signal was generated by using the SuperSignal West Pico Chemiluminescence Kit (Thermo Scientific) as described in the manufacturer’s manual. Stained membrane was imaged with an ImageQuant 350 camera (GE).

### Isolation and Purification of Total RNA

8-cell stage embryos were dissected by hand with the help of needles and a dissecting knife. Single blastomeres were collected and stored in 0,5 ml Trizol reagent (Invitrogen) to prevent RNA degradation by ribonucleases and homogenized by vortexing. Whole embryos were homogenized with a pestle in the Trizol reagent. Homogenized tissue was incubated at room temperature for five minutes. 0,1 ml chloroform was added per 0,5 ml Trizol and tubes were vortexed for 30 sec. After 10 min. incubation at room temperature, samples were centrifuged at full speed for 15 min. (4°C centrifuge). Aqueous phase, containing RNA, was transferred into a new tube and 0,5 ml isopropanol per 0,5 ml Trizol used was added. RNA was precipitated for 10 min. at room temperature or stored in isopropanol at −20°C. Sample was centrifuged at full speed, 4°C, for 15 min to pellet precipitated RNA. The supernatant was carefully removed with a 200 µl micropipette and pellet was washed with 70–80% ice-cold ethanol. The RNA pellet was air dried and resuspended in 100 µl DEPC-treated water. Subsequently, the resuspended RNA was purified using the RNeasy Micro Kit (Qiagen) as described in the manufacturer’s manual and resuspended in 14 µl RNase-free water. RNA concentration was measured on a Nanodrop spectrophotometer. RNA integrity was analysed using the RNA 6000 Pico Chip Kit (Agilent). RNA was stored at −80°C.

### RNA Amplification, Labelling and Hybridization on the Microarray

Total RNA, isolated and analysed as described above, was amplified and labelled using the Low Input RNA Amplification Kit Plus (Agilent) as described in the manufacturer’s manual. Labelled cRNA was purified with the RNeasy Micro Kit as described in the kit’s manual. Concentration, fluorescent dye incorporation and quality control were analysed by using the “Microarray” protocol for Nanodrop ND-1000 spectrophotometer and the RNA 6000 Pico Chip Kit (Agilent). Microarray hybridization was performed on custom 8×15 k microarray slides as described in Agilent’s protocol “Two-colour microarray-based gene expression analysis”, v. 5.5 (2007). Washing was performed with stabilization and drying solution as described in the supplemental procedures of the same protocol.

### Custom Gene Expression Microarray, Scanning and Data Extraction

A custom design microarray was created using the eArray platform (Agilent, Santa Clara, California, USA). The control grid used for the 8×15 k microarray was IS-15744-8-V1_8×15 K_Gx_EQC_V20060608.

The design was based on two EST sequence libraries for *Parhyale hawaiensis*, one from Tübingen and one from Berkeley (Nipam Patel, personal communication). Each EST was represented by at least one 60-mer probe on the array, with a total of 14158 probes for 9067 targets. The GEO accession number of the microarray platform is GPL16208.

Hybridized arrays were scanned with a GenePix 4000B scanner (Axon) using the default settings of the scanner. Data extraction was performed with Agilent’s Feature Extraction software (v. 9.5) and the standard protocol for two-coloured microarrays.

### EST Assembly and Probe Mapping

The EST sequences from both libraries were screened for known vector sequences using NCBI VecScreen and assembled into larger contigs using Cap3 [38]. Of 9067 ESTs, 3650 were combined into 1040 contigs, 5392 remained as singletons. The probe sequences designed for the array were mapped against the resulting 6432 targets using RazerS [39]. To exclude influences of cross-hybridization, only uniquely mapping probes were further considered (11666). 5985 targets were assigned at least one probe, the majority (3228, 65%) were assigned two or more probes. 1020 probes were mapped to more than one target and were discarded from further analysis. In a second round of mapping the non-uniquely matched probes were mapped to all ESTs (to allow for the possibility that probes match to parts of ESTs trimmed during contig assembly). This resulted in the addition of further 620 probes to the analysis and the extension of some existing contigs (therefore 376 contigs appear twice in the analysis and the redundant entries were labelled with “ext”, e.g. ContigXXX_ext). In total, 13264 probes for 6386 targets were used for the analysis (11682 probes corresponding to the 6361 EST-based targets and 1175 probes corresponding to 25 genes of interest).

### Background Correction, Data Normalization and Processing

The first background correction was done during feature extraction using the standard setting of the manufacturer’s Feature Extraction software. The rest of the data evaluation was done using the limma package from Bioconductor [40], an open source software for bioinformatics running in the R environment (R Development Core Team, *R: A Language and Environment for Statistical Computing*, http://www-R-project.org ).

Probe-level data from the arrays were background-corrected using the ‘normexp’ method with an offset of 50, technical variation due to dye-dependent effects was addressed by printtip-loess normalization and the resulting data were normalized between arrays using the ‘Aquantile’ method. This has the effect to remove array-dependent variations in signal strength, ensuring that the mean intensity distributions of all arrays are the same.

Values from multiply spotted identical probes (replicates) were averaged using the mean. Based on the assignment of probes to targets, the intensities of the probes assigned to each contig were summarized using the robust median polish method (R script and data analysis algorithm available upon request).

### Statistical Analysis

The moderated t-statistics, moderated F-statistic, and log-odds of differential expression were computed by empirical Bayes (eBayes) shrinkage of the standard errors towards a common value (1% differentially expressed genes) [41]. The Benjamini-Yekutieli method was applied to correct for multiple testing.

The three experiments (Maternal/Zygotic, Ectoderm, Mesoderm) each consisted of multiple biological and technical replicates (eight replicates for each experiment, at least two biological), some of which were reverse labelled (dye swap). This information is specified by the design parameters and used for the linear model fit of the data, generating the average log_2_ fold change, standard error and log_2_ expression level for each target.

The identification of differentially distributed transcripts between Ectoderm and Mesoderm was based on the sets of statistically significant targets (p<0.05). The evaluation of the “Maternal vs. Zygotic” experiment was based on a significance threshold of p<0.05.

Further analyses and visualizations were done with Mayday [42].

### Microarray Layout

We performed three groups of experiments, all with biological and technical replicates. The samples used as technical replicates were split prior to hybridization on the chip. A total of 24 microarrays were used, distributed on four 8×15 k microarray slides (Figure S1). The biological material was obtained after microdissection of single embryos, which was a limiting factor. Therefore in some cases the input material was only enough for two biological replicates and two technical replicates; in these cases we filled up the remaining arrays on each slide with more technical replicates of biological samples which were converted into labelled cRNA more efficiently. For the “Early vs. Late” experiments, four biological replicates were used, and each of the biological replicates was hybridized to two microarrays (technical replicates). After quality control during the feature extraction step, two arrays were discarded, leaving a total of three biological replicates on six arrays (2+2+2). For the “Ectoderm vs. Rest” experiments, four biological replicates were used, two of them in two technical replicates, one in three technical replicates, and one on a single array. After discarding one array, a total of seven arrays (2+2+3) remained for the data analysis. For the “Mesoderm vs. Rest” experiments, two biological replicates were used and hybridized to a total of eight (2+6) microarrays. Fold-change values, differential expression and statistical significance were computed using all high-quality replicate values for each condition (after discarding low-quality arrays, see above). Furthermore, the algorithm that we used for the data analysis, Limma, differentiates between technical and biological replicates, which compensates for the different number of technical replicates. The raw and processed data from the microarray experiments is available at GEO (package accession number GSE41888).

### Annotation and Gene Ontology Enrichment Analysis

The 6542 targets were annotated using the blast2go platform and NCBI’s BLAST [21],[43]. GO enrichment analysis was performed also in blast2go using Fisher’s Exact Test with a cut-off for the p-value of 0.05.

### Sequence Analysis

Primer design and protein sequence alignments were done by using MacVector v. 8.0 (Accelrys). Overlapping sequences were analysed using AssemblyLIGN v. 1.09 (DNASTAR). Restriction enzyme sites in DNA sequences were analysed using NEB Cutter 2.0. In all cases the default settings of the manufacturer were used.

## Supporting Information

Figure S1 **Microarray layout.** Schematic representation of the four 8×15 k microarray slides used for the three groups of experiments performed in this study. Each box corresponds to a single two-color 15 k-array. For each array we used a pair of biological samples (e.g. “Ect” = pool of ectoderm progenitor cells; “Rest” = the remaining blastomeres from the same microdissection of embryos), labeled in parallel with either Cy5 or Cy3 fluorescent dyes. Numbers in the name of the samples indicate biological replicates. The correlation coefficient (R-squared value) for each array was calculated by Agilent’s Feature Extraction software based on the manufacturer’s control probes. Arrays that have a low R-squared value have been discarded from the analysis (dashed line). We used eight arrays for experiments not related to the current study (grey boxes).
(TIF)

Figure S2 **Correlation matrix plots for 8-cell stage microarray datasets.** For generating the correlation matrix we used the signal intensity values (processed signal after subtraction of the background intensity, generated by Agilent’s Feature Extraction software and prior to normalization). The heatmap illustrates the Spearman’s rank correlation coefficient for (A) the “Ectoderm vs. Rest” and (B) the “Mesoderm vs. Rest” datasets. The sample names correspond to the ones used in Tables S2 and S3.
(TIF)

Figure S3 **Semi-quantitative PCR for Ph-cg1295 siRNA injected embryos.** The RNAi knock-down experiment was validated by showing downregulation of the target RNA in siRNA-injected embryos. The semi-quantitative PCR was performed on three different cDNA templates, which were prepared from the RNA of embryos injected with Stealth-RNA1, Stealth-RNA2 and DEPC-water (Control injection) respectively. The embryos were injected at the 1-cell stage and RNA was isolated at the 8-cell stage. Beta-actin (Ph-b-act) was used as a reference gene.
(TIF)

Table S1 **Microarray analysis of early vs. late embryonic transcriptomes.** Full dataset from the transcriptome analysis of early (1- to 4-cell stage embryos) vs. late (embryonic day 2 to day 5) *Parhyale* embryos. The “Description” sheet of the Excel file contains an explanation of the table headers, as well as a list of samples and brief summary of the experimental procedure.
(XLSX)

Table S2 Microarray analysis of the 8-cell stage: ectoderm progenitors vs. rest of the embryo. Full dataset from the lineage-specific transcriptome analysis of the 8-cell stage embryo, where pools of ectoderm progenitor cells were compared to the remaining blastomeres. The “Description” sheet of the Excel file contains an explanation of the table headers, as well as a list of samples and brief summary of the experimental procedure.
(XLSX)

Table S3 Microarray analysis of the 8-cell stage: mesoderm progenitors vs. rest of the embryo. Full dataset from the lineage-specific transcriptome analysis of the 8-cell stage embryo, where pools of mesoderm progenitor cells were compared to the remaining blastomeres. The “Description” sheet of the Excel file contains an explanation of the table headers, as well as a list of samples and brief summary of the experimental procedure.
(XLSX)

Table S4 Summarized results for the 129 RNAs distributed asymmetrically in the 8-cell stage embryo. A list of the 129 unique RNAs that were identified as enriched in a subset of blastomeres at the 8-cell stage *Parhyale* embryo. The table provides gene annotation, as well as the fold-change and adjusted p-values from the three microarray experiments (as in tables S1–S3) for each of the 129 RNAs. The “Description” sheet of the Excel file contains an explanation of the table headers.
(XLSX)

Table S5 Gene ontology enrichment analysis. GO term analysis for each of the three microarray experiments (based on the processed and annotated results as given in tables S1–S3). The “Description” sheet of the Excel file contains an explanation of the table headers.
(XLSX)

Table S6 Summarized results of ISH experiments. ISH analysis was performed for 10 RNAs identified by microarray as differentially distributed at the 8-cell stage. The table provides a summary of the observed results, as well as the fold-change values from the microarray experiments (as in tables S2–S3). The “Description” sheet of the Excel file contains an explanation of the table headers.
(XLSX)
