# Supplementary figures and images for: The Maternal Transcriptome of the Crustacean Parhyale hawaiensis Is Inherited Asymmetrically to Invariant Cell Lineages of the Ectoderm and Mesoderm
Source: PLoS One. 2013 Feb 13;8(2):e56049. doi: 10.1371/journal.pone.0056049 (PMC3572164; doi:10.1371/journal.pone.0056049)

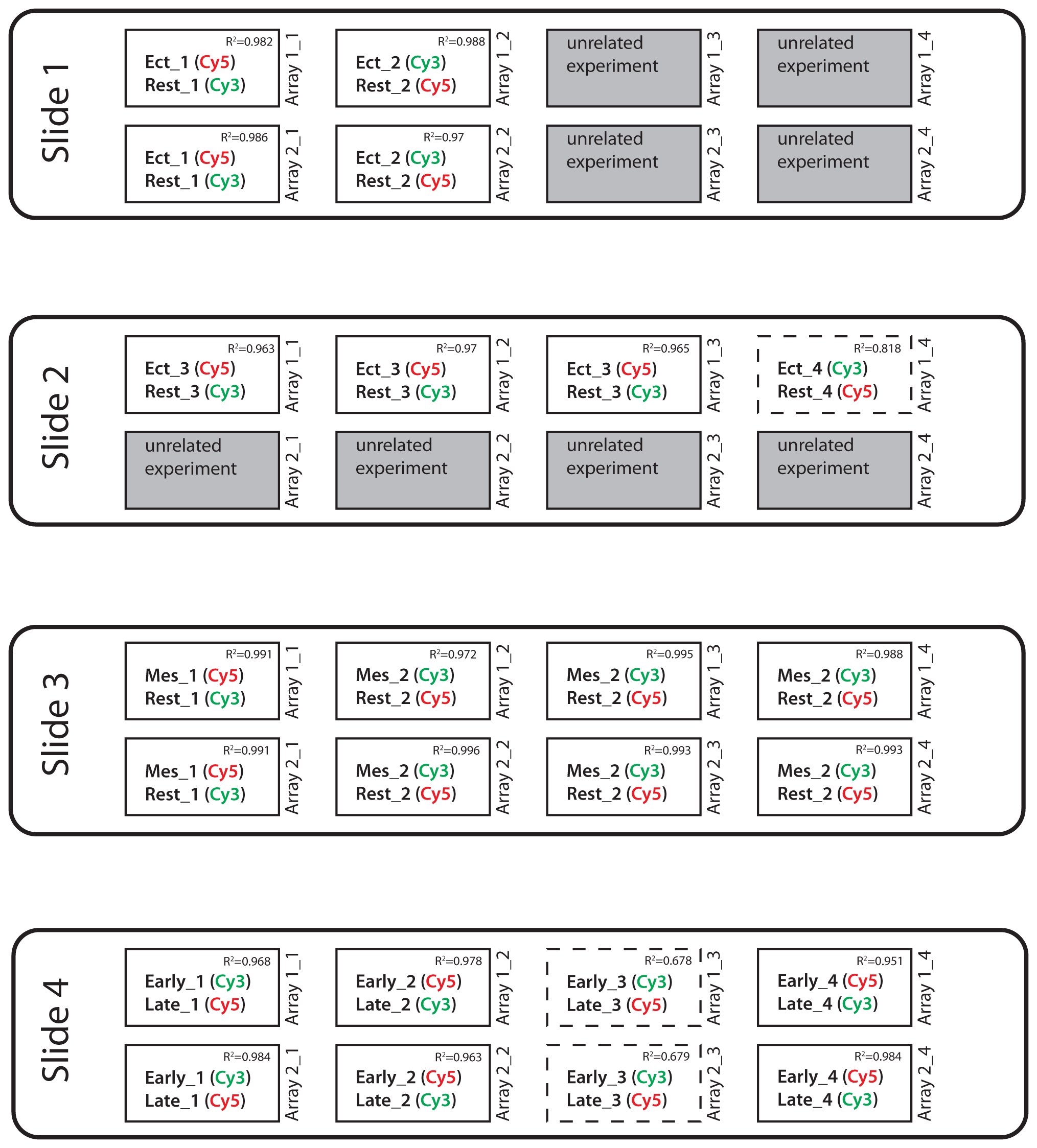

Supplement: Figure S1 — Microarray layout. Schematic representation of the four 8×15 k microarray slides used for the three groups of experiments performed in this study. Each box corresponds to a single two-color 15 k-array. For each array we used a pair of biological samples (e.g. “Ect” = pool of ectoderm progenitor cells; “Rest” = the remaining blastomeres from the same microdissection of embryos), labeled in parallel with either Cy5 or Cy3 fluorescent dyes. Numbers in the name of the samples indicate biological replicates. The correlation coefficient (R-squared value) for each array was calculated by Agilent’s Feature Extraction software based on the manufacturer’s control probes. Arrays that have a low R-squared value have been discarded from the analysis (dashed line). We used eight arrays for experiments not related to the current study (grey boxes). (TIF) [file pone.0056049.s001.tif]

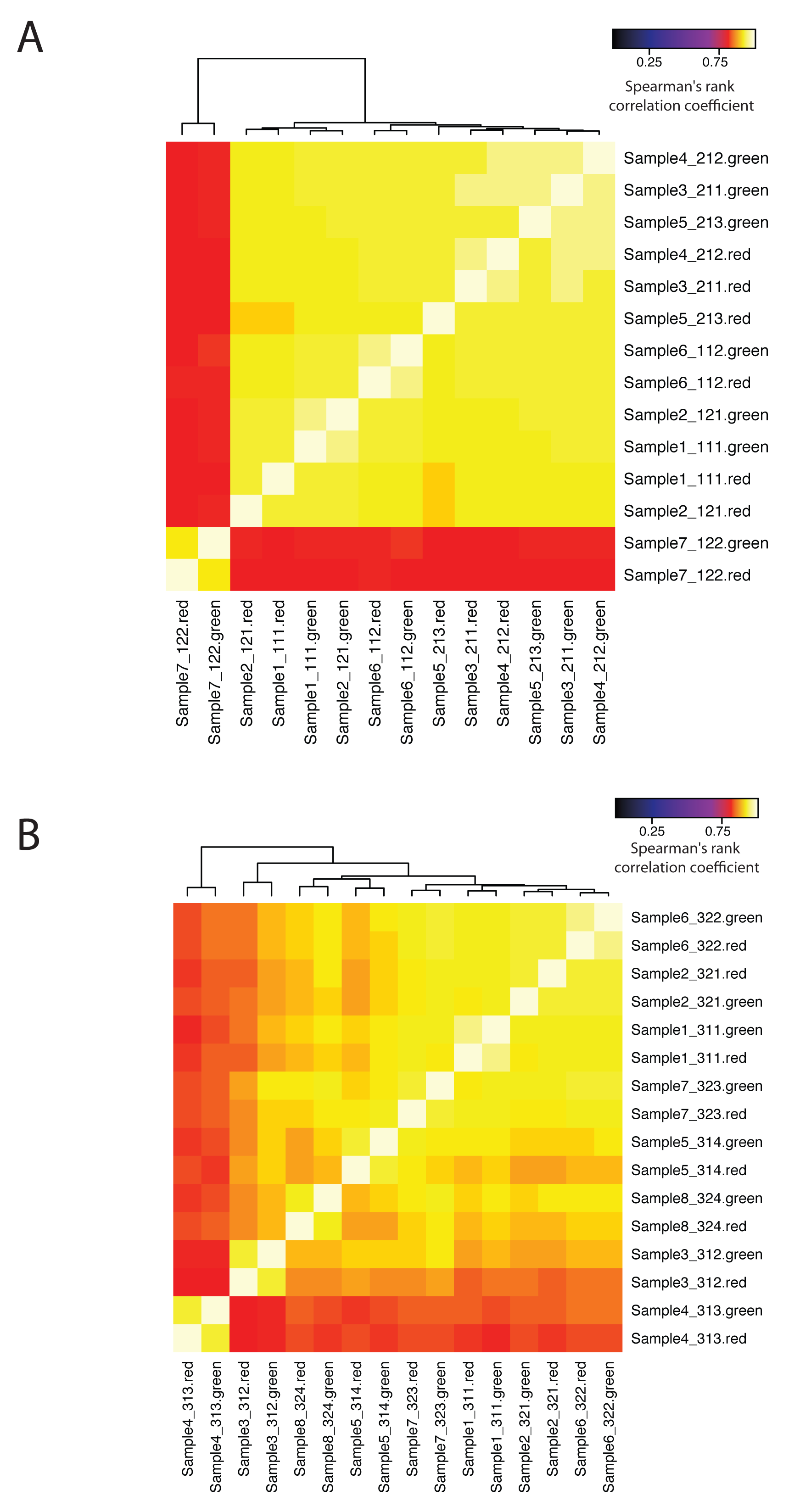

Supplement: Figure S2 — Correlation matrix plots for 8-cell stage microarray datasets. For generating the correlation matrix we used the signal intensity values (processed signal after subtraction of the background intensity, generated by Agilent’s Feature Extraction software and prior to normalization). The heatmap illustrates the Spearman’s rank correlation coefficient for (A) the “Ectoderm vs. Rest” and (B) the “Mesoderm vs. Rest” datasets. The sample names correspond to the ones used in Tables S2 and S3. (TIF) [file pone.0056049.s002.tif]

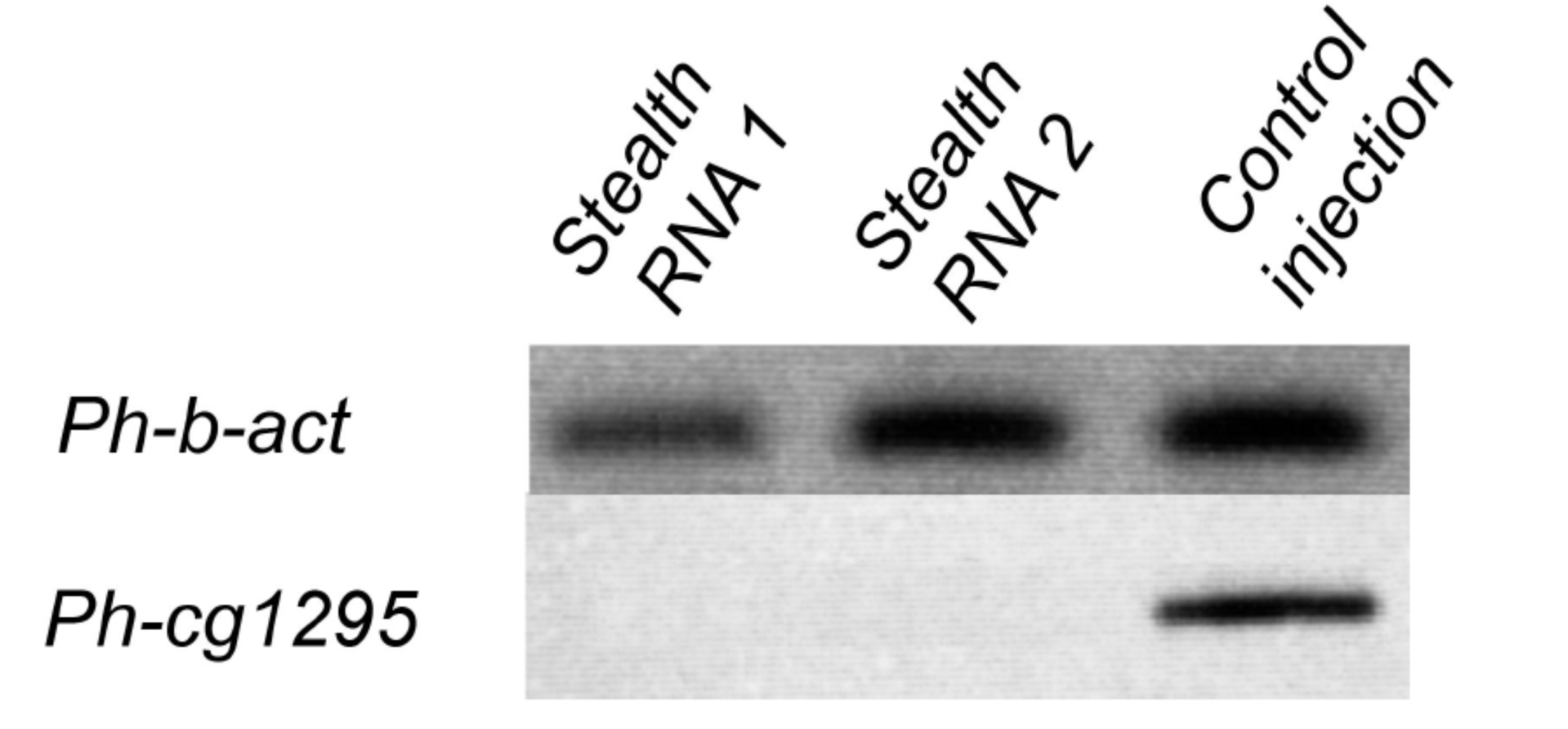

Supplement: Figure S3 — Semi-quantitative PCR for Ph-cg1295 siRNA injected embryos. The RNAi knock-down experiment was validated by showing downregulation of the target RNA in siRNA-injected embryos. The semi-quantitative PCR was performed on three different cDNA templates, which were prepared from the RNA of embryos injected with Stealth-RNA1, Stealth-RNA2 and DEPC-water (Control injection) respectively. The embryos were injected at the 1-cell stage and RNA was isolated at the 8-cell stage. Beta-actin (Ph-b-act) was used as a reference gene. (TIF) [file pone.0056049.s003.tif]
